# Micro-Fabricated Resonator Based on Inscribing a Meandered-Line Coupling Capacitor in an Air-Bridged Circular Spiral Inductor

**DOI:** 10.3390/mi9060294

**Published:** 2018-06-12

**Authors:** Eun Seong Kim, Nam Young Kim

**Affiliations:** Radio Frequency Integrated Centre (RFIC), Kwangwoon University, Kwangwoon-ro, Nowon-gu, Seoul 01897, Korea; esk@kw.ac.kr

**Keywords:** air-bridge, bandpass filter, meandered-line coupling capacitor, micro-fabricated, spiral inductor

## Abstract

This letter presents a high-performance micro-fabricated resonator based on inscribing a meandered-line square coupling capacitor in an air-bridged circular spiral inductor on the GaAs-integrated passive device (IPD) technology. The main advantages of the proposed method, which inserts a highly effective coupling capacitor between the two halves of a circular spiral inductor, are the miniaturized size, enhanced coupling coefficient, and improved selectivity. Moreover, using an air-bridge structure utilizes the enhanced mutual inductance in which it maximizes the self-inductance by a stacking inductor layout to obtain a high coupling effect. The simulated and measured S-parameters of a prototype resonator with an effective overall circuit size of 1000 µm × 800 µm are in good agreement. The measured insertion and return losses of 0.41 and 24.21 dB, respectively, at a measured central frequency of 1.627 GHz, as well as an upper band transmission zero with a suppression level of 38.7 dB, indicate the excellent selectivity of the developed resonator.

## 1. Introduction

Global positioning system (GPS) receivers are being miniaturized and their cost is being minimized to make them accessible for virtually everyone. This has tremendously increased the demand for high-performance compact resonators. An increased growth in the publication rate on microstrip lines and printed circuit board (PCB) technology-based resonators partially reflects the intense interest that resonators for GPS receivers have been generating [1,2,3]. However, PCB-technology-based resonators still suffer from many bottlenecks, such as limited design flexibility, because of the impracticalities in implementing three-dimensional structures, large critical dimensions (~0.2 mm), and large fabrication tolerances. Therefore, they cannot fulfil the demands of high-level circuit miniaturization. On the other hand, integrated passive device (IPD) technology provides noticeably improved critical dimensions (~5 µm), extended design flexibility with air-bridge structures, low fabrication tolerances, and the capability to integrate with active circuits.

Therefore, IPD-based implementation of high-performance, miniaturized resonators has recently garnered an increased amount of interest from researchers [4,5,6]. IPDs typically combine a number of passive components into a single package. These devices are increasingly being built using GaAs thin-film substrates rather than on silicon-like semiconductor devices or ceramic, which have very poor characteristics or can be difficult to tune. The integration of a large number of passive components including the individual passive devices such as transmission lines, inductors, and capacitors, or functional passive devices with low loss and minimal crosstalk, is as important as the advancement of active transistor technology [7,8]. IPDs are 50% smaller, 70% thinner, and require 50% less printed circuit board (PCB) area compared to discrete passive devices. In addition, IPDs are a bridge platform between the front and back ends of the semiconductor process. The use of air-bridged differential transformers to miniaturize the effective size of the resonators has been gaining much attention owing to the high self-inductance and increased mutual inductance of such transformers [9]. Metal–insulator–metal (MIM) capacitors with optimized plate areas can be embedded between the two halves of the differential transformers to obtain highly miniaturized, high selectivity resonators [10,11]. However, the requirement of a SiNx dielectric layer increases the fabrication complexity and cost. Other manufacturing challenges include materials and process standardization, library component development, correlation between the design and IPD performance, and integration of the IPD. Also, a higher capacitor density at a lower manufacturing cost is critical [12].

This study reports on the development of a compact resonator based on a micro-fabricated circular spiral inductor and meandered-line coupling capacitor. The noticeable advantages of size miniaturization and selectivity improvement, which are attributed to the coupling capacitor and verified by the S-parameter and derived-lumped-parameter–based analyses, validate the importance of the proposed method in developing high-performance resonators. The measured excellent selectivity of the developed resonator’s insertion and return losses of 0.41 and 24.21 dB, respectively, at a measured central frequency of 1.627 GHz, as well as an upper band transmission zero with a suppression level of 38.7 dB, shows the excellent selectivity in the developed resonator.

## 2. Materials and Methods

### Resonator Design

A planar, square, meandered-line coupling capacitor was inscribed in a circular spiral inductor to produce the proposed resonator. The coupling capacitor was inserted in series between the two symmetric halves of the spiral inductor. Therefore, the produced resonator is equivalent to a series combination of its distributed parameters including the loss resistance (*R*), net inductance (*L_CS_*), and coupling capacitance (*C_C_*) [13]. Consequently, the resonator generates a central frequency (*f*_0_) expressed by the following equation:

(1) $$f_{0}=\frac{1}{2\pi }\sqrt{\frac{1}{L_{CS}C_{C}}-{\left(\frac{R}{L_{CS}}\right)}^{2}}$$

**Figure 1:**
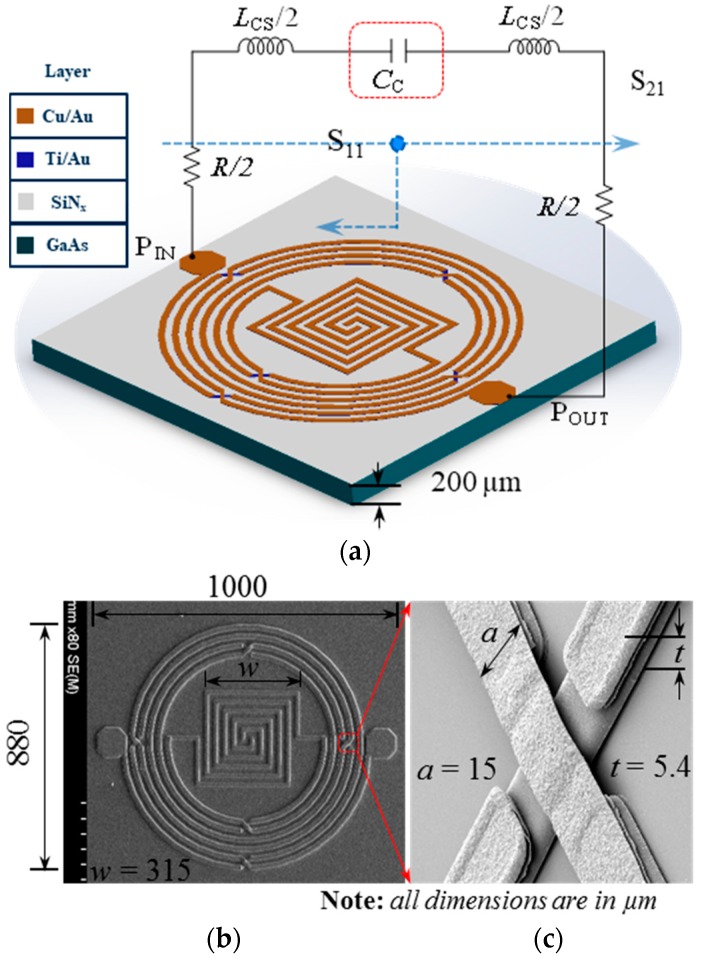
Proposed resonator based on a micro-fabricated air-bridged circular spiral inductor and meandered-line coupling capacitor. (**a**) 3D layout and equivalent lumped-element circuit; (**b**) scanning electron microscopy (SEM) image of fabricated resonator; and (**c**) enlarged focused ion beam (FIB) image of the air-bridge structure.

The proposed resonator based on a micro-fabricated air-bridged circular spiral inductor and meandered-line coupling capacitor is shown Figure 1. The 3D layout and equivalent circuit of the resonator (a), a scanning electron microscopy (SEM) image of the fabricated resonator (b), and an enlarged focused ion beam (FIB) image of an air-bridge structure is shown in (c). In the (a) image, GaAs substrate (400 μm) was used to allow high-speed microelectronics. The first passivation layer deposited was SiNx at 2000 Å thickness. Next, a seed metal layer of Ti/Au at 20/80 nm thickness was deposited. Lastly, Cu at a thickness of 3.35 μm was formed. According to the expression based on a current-sheet expression [14], the inductance of a planar circular spiral inductor can be expressed as follows:

(2) $$L_{CS}=\frac{\mu_{0}n^{2}d_{\mathrm{avg}}c_{1}}{2}\left(1n\left(c_{2}/\rho \right)+c_{3}\rho +c_{4}\rho^{2}\right)$$

where *µ*_0_ (= 4π × 10^−7^ H/m) denotes the space permeability and *n* is the number of turns. *c*_1_, *c*_2_, *c*_3_, and *c*_4_ are layout-dependent coefficients and exhibit values of 1, 2.46, 0, and 0.2, respectively, for a circular layout. *ρ* = (*d*_out_ − *d*_in_)/(*d*_out_ + *d*_in_) and *d*_avg_ = (*d*_out_ + *d*_in_)/2 denote the fill ratio and average diameter, respectively, in terms of the outer diameter (*d*_out_) and inner diameter (*d*_in_). The corresponding metal traces were crossed over using the air-bridge structure, to utilize the enhanced mutual inductance, and the lower metal layer was used as a bridge. A stacked inductor layout was employed to maximize the self-inductance and achieve high area efficiency for a high coupling coefficient [15].

The net coupling capacitance (*C_C_*) between the coupled lines can be expressed as follows:

(3) $$C_{C}=\left[\varepsilon_{0}\left(\frac{1+\varepsilon_{s}}{2}\right)\frac{K\left(\sqrt{1-k^{2}}\right)}{K\left(k\right)}+\varepsilon_{0}\frac{t}{a}\right]L_{C}$$

where *ε*_0_ and *ε*_s_ represent the dielectric constants of air and the substrate, respectively. *L_C_* denotes the total length of the coupled lines and *k* (= *t*/*a*) and *K*(*k*) represent the elliptical integral of the first kind.

(4) $$Q=\frac{f_{0}}{B_{3‐dB}}\vdots B_{3‐dB}=f_{h}-f_{l}$$

(*Q*) is the quality factor and (*f*_0_) is the center frequency. (*B*_3-*d*B_) is the 3-*d*B bandwidth. The meandered-line coupling structure is used to maximize the coupling length and, therefore, the coupling capacitance in a minimum area [16].

## 3. Results and Discussion

**Figure 2:**
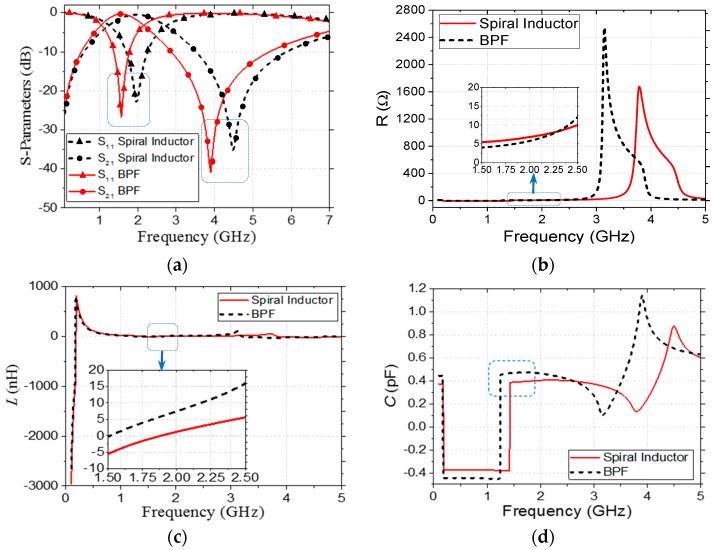
S-parameters and lumped parameters to study the effect of a coupling capacitor. (**a**) Simulated S11 and S21; (**b**) resistance (*R*); (**c**) inductance (*L*); and (**d**) capacitance (*C*).

A typical resonator based on the proposed design layout was simulated and optimized using Agilent Advanced Design System (ADS) software (version 2016.01, Keysight Technologies, Inc., Santa Rosa, CA, USA) to generate a central frequency of 1.574 GHz. The optimized dimensions were *d*_in_ = 530 µm, *d*_out_ = 880 µm, and *L_C_* = 1850 µm. The air-bridged circular inductor consisted of five turns (*n* = 5) with a 15 µm gap between the corresponding turns. The simulated S-parameters, which are shown in Figure 2a, indicate that the air-bridged inductor alone resonated at a central frequency of 1.98 GHz and exhibited a 5 GHz transmission zero with 22.9 dB suppression. The capacitive effect required that the resonance be provided by the coupling between the top metal layer and the ground aluminum box through the sandwiched GaAs substrate, and has been shown in the detailed equivalent circuit in our previous work [17]. In Figure 2a–d, the spiral inductor graph indicates a circular spiral inductor that is outside of the design and a bandpass filter (BPF) in the inner meandered-line square capacitor. Additionally, the inscribing of the meandered-line coupling capacitor shifted the central frequency downward to 1.574 GHz and, therefore, reduced the effective size of the resonator by 26.67%.

**Figure 3:**
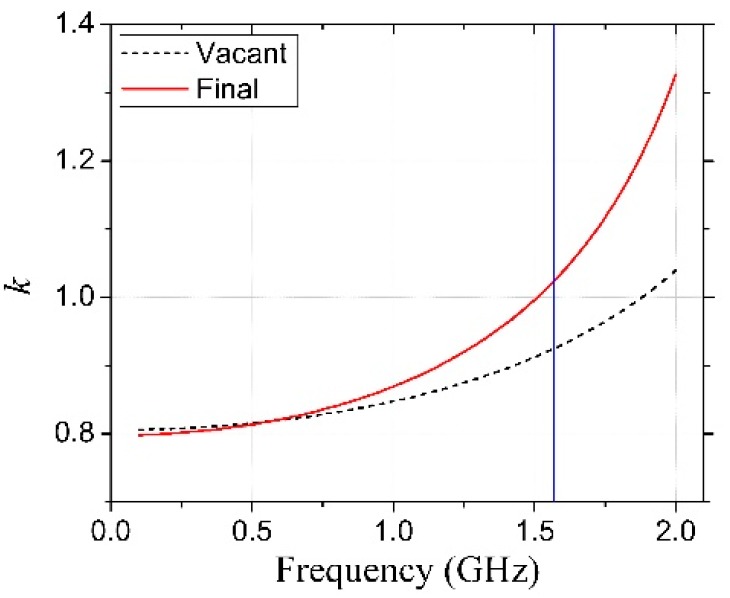
Variation of the coupling coefficient (*k*) with frequency for an air-bridged spiral inductor alone and for the proposed resonator.

**Figure 4:**
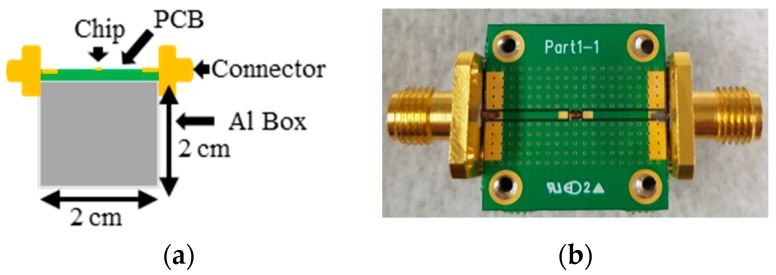
A printed circuit board (PCB) and ground are shown in (**a**) cross-section view; (**b**) and a top section with a design chip.

Moreover, it markedly enhanced the passband return loss and transmission zero suppression level and, therefore, improved the resonator selectivity. The distributed resistance (*R*), inductance (*L_CS_*), and capacitance (*C_C_*) were extracted from the simulated S-parameters [18] to analyze the effect of the coupling capacitor on the resonator performance, and the corresponding results are shown in Figure 2. It was observed that the use of the meandered-line structure increased *L_CS_* (from 1.09 nH to 4.21 nH) and *C_C_* (from 0.38 pH to 0.47 pH) slightly and decreased *R* (from 5.57 Ω to 4.22 Ω) at 1.574 GHz. In addition, the calculated coupling coefficient (*k*), which is shown in Figure 3, indicated that the use of the coupling capacitor generated an over-coupled (*k* = 1.04) resonator at 1.574 GHz. A prototype resonator with optimized dimensions was fabricated on a conventional 6-inch, 200-μm-thick GaAs substrate with a dielectric constant ε_s_ = 12.85 and loss tangent tan δ = 0.006; the detailed fabrication process is explained elsewhere [19,20]. The coupling capacitor was realized with *k* = 0.36 and *K* = 1.6257. Figure 1b,c illustrates a magnified scanning electron microscopy (SEM) image of the fabricated resonator with overall physical dimensions of 1000 µm × 880 µm and a focused ion beam (FIB) image of the air-bridge structure, respectively [21]. Figure 4 shows that the PCB board was attached to a 2 cm^2^ Al Box as a ground to decrease the noise. Both sides were connected to an subminiature version A (SMA) connector to measure with a vector network analyzer (VNA).

**Figure 5:**
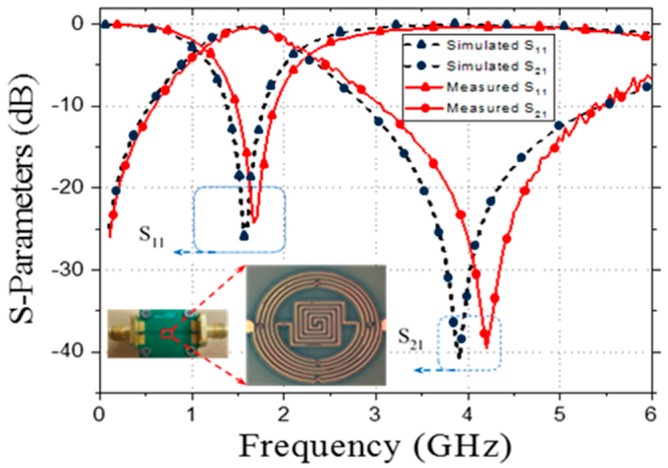
Simulated and measured S-parameters and the pictures of the fabricated resonator.

The S-parameters measured using an Agilent 8510C VNA demonstrated good agreement with the simulated values. However, a small (53 MHz) downward shift of the measured central frequency (1.627 GHz) was observed with respect to the simulated value [22]. The measured 3 dB fractional bandwidth of the passband was 54.08%. The small differences in the central frequency and fractional bandwidth could be because of the substrate dielectric loss, dispersion loss at the inductor bends, and the accuracy of the physical dimensions. Figure 5 shows that the measured insertion and return losses of the passband were 0.41 and 24.21 dB, respectively. A transmission zero appeared at 3.9 GHz with a high suppression level of 38.7 dB. The distributed parameters at 1.627 GHz, extracted from the measured S-parameters, were *R* = 4.45 Ω, *L_CS_* = 1.08 nH, and *C_C_* = 0.48 pF [23].

Table 1, which compares our developed resonator with several recently reported IPD resonators, indicates that our work demonstrates a high-performance resonator with a more compact size and better selectivity owing to the lower insertion loss and higher return loss. In addition, the smaller number of metal layers reduces the fabrication complexity and cost of the device.

## 4. Conclusions

A highly miniaturized resonator based on an air-bridged circular spiral inductor and an inscribed square meandered-line coupling capacitor was developed using GaAs-based micro-fabrication IPD technology. Lumped-parameter-based analysis revealed that a system of meandered coupled lines between the two halves of a circular spiral inductor could provide high coupling capacitance, miniaturize the effective size, and improve the selectivity of the resulting resonator, significantly. The measured excellent selectivity of the developed resonator’s insertion and return losses of 0.41 and 24.21 dB, respectively, at a measured central frequency of 1.627 GHz, as well as an upper band transmission zero with a suppression level of 38.7 dB, shows a good candidate for use in the next upgrade of GPS applications.

## Figures and Tables

**Table 1:** Performance Comparison with Reported Resonators.

Reference	Technology	CF ^1^ (GHz)	IL ^2^ (dB)	RL ^3^ (dB)	Size (mm^2^)	Metal Layers
[17]	GaAs IPD	2.27	0.8	26.1	0.9	2
[24]	Silicon IPD	2.45	2.2	30	1.5	3
[25]	GaAs IPD	7.7	1.63	40.1	4.98	1
This work	GaAs IPD	1.627	0.4	24.2	0.88	2

^1^ Central Frequency, ^2^ Insertion Loss, ^3^ Return Loss.
